# Diagnosis, management, and outcomes of non-traumatic gastrointestinal tract perforations in A rural district hospital in Northern Cameroon: a retrospective observational study

**DOI:** 10.3389/fsurg.2026.1962233

**Published:** 2026-10-30

**Authors:** Ubraine Njineck Wunde, Wesley Harrisson Bouche Djatche, Dimitri Tchinda, Gaetan Konfo Kwasseu, Arafat Boubadjam, Victor Meza Kyaruzi

**Affiliations:** 1 Department of Surgery, Hôpital Privé Catholique de Tokombéré, Maroua, Cameroun; 2 Department of Surgery, Presbyterian Health Complex Nsimeyong Yaounde, Yaounde, Cameroon; 3 Research Division, Winners Foundation, Yaounde, Cameroon; 4 Faculty of Medicine and Biomedical Sciences, University of Yaounde 1, Yaounde, Cameroon; 5 Faculty of Health Sciences, University of Buea, Buea, Cameroon; 6 Department of Surgery, School of Medicine, Muhimbili University of Health and Allied Sciences, Dar es Salaam, Tanzania

**Keywords:** Cameroon, emergency laparotomy, gastrointestinal perforation, Maroua, peritonitis

## Abstract

**Background:**

The gastrointestinal tract is vulnerable to perforations across multiple segments, with diverse etiologies. While existing research has largely focused on urban hospitals, data from rural settings remain scarce. This study aims to determine the frequency of non-traumatic gastrointestinal perforations in a rural, hard-to-reach district hospital in Northern Cameroon, to describe the local management approaches and address gaps in rural emergency surgical care delivery.

**Methods:**

We conducted a retrospective descriptive study of patients who underwent surgery for non-traumatic gastrointestinal tract perforation at Tokombéré District Hospital from January 2021 through January 2023. Categorical variables were compared using Pearson’s Chi-square test or Fisher’s exact test, and continuous variables using the Mann–Whitney U or Kruskal–Wallis test. Significance was set at *p* < 0.05.

**Results:**

A total of 44 patients were included, with a median age of 21.5 years (IQR 9-43). Abdominal pain was the predominant presenting symptom (70.5%, 31/44), and abdominal x-ray served as the main diagnostic tool (36.4%, 16/44). The median time interval from symptom onset to definitive surgery was 6 days (IQR 4–10). The small bowel emerged as the most frequent site of perforation (47.6%), and simple repair was the primary treatment modality (76.3%). Postoperative complications occurred in 27 patients (61.4%), and postoperative in-hospital mortality was 4.5% (2/44).

**Conclusion:**

The rural district hospital setting in Northern Cameroon imposes particular challenges to the diagnosis, management and follow-up of nontraumatic gastrointestinal perforations. Delayed presentation is a common finding, and children and young adults accounted for most cases in this cohort.

## Introduction

Gastrointestinal tract (GIT) perforations can occur across various segments of this hollow viscus, caused by different aetiologies. In many developing countries, the majority of these perforations are non-traumatic, representing one of the most frequent causes of peritonitis (1–3). However, aetiologies differ between developed and developing settings: tumoral causes are more common in the developed world, while infectious causes (Typhoid, Tuberculosis) predominate in the developing world. There has, however, been a gentle drift in these trends over time (4), with the developing world facing a double burden of disease.

Despite the severity of this burden in rural areas, most studies on this pathology are focused on trends in the urban areas served by tertiary and quaternary healthcare facilities (1, 5, 6). While the causes of peritonitis are wide and vary from primary through secondary to tertiary forms (1), this study focuses specifically on secondary peritonitis resulting from non-traumatic causes. Typhoid fever, endemic in sub-Saharan Africa remains a commonly reported aetiology of GIT perforations in this region (3, 7). Despite sparse data on its actual prevalence, recent African series show that perforations account for a large share of operated peritonitis: in Burkina Faso, 120 of 145 patients (82.8%) operated for acute generalised peritonitis over a one-year period at the Ouahigouya Regional University Hospital had a GI perforation (8), and in Mali, non-traumatic digestive perforations accounted for 61 of 78 cases of acute peritonitis (78.2%) managed at the Koutiala Reference Health Centre over 17 months (7). In Cameroon, Ngo Nonga et al. reported 30 digestive perforations among 69 patients (43.5%) operated for peritonitis at the Yaoundé University Teaching Hospital (9), while Savom et al., in a multicentre study, identified 52 patients with spontaneous terminal ileal perforation over five years (2017–2021) in five referral hospitals in Yaoundé and Douala, i.e., approximately 10 cases per year (5). These studies narrow down towards a similar conclusion; gastrointestinal perforations are a growing medico-surgical emergency and the setting has an important influence on the aetiology and hence management.

The diagnosis of GIT perforations rests on the clinical presentation, corroborated by abdominal imaging. Moreover, the management is principally surgical with the need for urgent bespoke surgical intervention. This is sometimes not readily available at the primary healthcare level. The specific surgical approach and procedures depend on a myriad of factors; the site of the perforation and the patient’s hemodynamic status at presentation. Due to systemic limitations within the regional healthcare infrastructure in Cameroon, patients frequently present with advanced disease (1, 7), making management in hard-to-reach areas even more challenging.

Consequently, while prompt diagnosis and timely surgical intervention are indispensable to curb the morbidity and mortality of this rapidly progressing pathology, there remains a critical lack of data regarding current management practices and outcomes in resource-limited, primary health care settings. By shifting the focus, this study aims to address this data gap and highlight the realities of managing GI perforations in a rural setting of Northern Cameroon.

## Study objectives

Our objective is to: 1) Describe the clinical presentation and diagnostic approaches of non-traumatic GIT perforations at a District Hospital in Tokombere, Northern Cameroon; and 2) Describe the treatment approaches and outcomes in patients with non-traumatic GIT perforations at a District Hospital in Tokombere, Northern Cameroon.

## Methods

### Study design and period

This was a retrospective descriptive study of patients managed for non-traumatic GIT perforations at the surgical department of the Private Catholic Hospital of Tokombere (Tokombere District Hospital) from January 2021 to January 2023.

### Study area and setting

This study was conducted in the surgical department of the aforementioned hospital, located in the rural areas of the extreme north region of Cameroon. It is noted for its hard-to-reach nature during the rainy season, with an average distance from the regional hospital being 48.2 km equal to about 3 h ride in fair weather. The main method of patient transportation in this area is by motorcycle. This hospital is recognised as the district hospital for Tokombere with a capacity of 152 beds, three general practitioners with surgical skills, no trained anesthesiologist and 5 surgical care nurses. It serves as a referral hospital for 13 integrated health centres and provides care to over 100 neighbouring villages, which constitute the catchment area for our patient base. The surgical department comprises a surgical ward with a capacity of 40 beds, a theatre, and a modern sterilisation unit.

### Study population

We included all files of patients with the diagnosis of non-traumatic gastrointestinal tract perforations aged 1 month and above (i.e., ≥ 28 days; neonates were therefore not eligible) who had an initial surgical intervention performed in the surgical departments of the aforementioned hospital within the stated time frame.

We excluded patients with missing data on both management and outcomes, patients who died before initiation of management, and patients with a traumatic GIT perforation. Patients who died before reaching the hospital, were managed non-operatively, were treated in another facility or were transferred out before surgery could not be captured by this design.

Case definition: a non-traumatic GIT perforation was defined as a full-thickness defect of the wall of the gastrointestinal tract (stomach, duodenum, small bowel, appendix, colon or rectum) occurring in the absence of blunt or penetrating abdominal trauma. The diagnosis was suspected preoperatively on clinical grounds (acute abdominal pain with signs of local or generalised peritoneal irritation) supported by imaging (pneumoperitoneum on plain abdominal x-ray and/or free intraperitoneal fluid on ultrasound), and was confirmed intraoperatively in every included patient by direct visualisation of the perforation, as documented in the operative report. Aetiological classification: blood culture, typhoid serology and histopathological examination were not available in this setting, so the underlying cause of perforation could not be confirmed in any patient. Perforations were therefore classified by anatomical site and number of perforations as recorded in the operative report; any aetiological attribution (for example, typhoid fever for ileal perforations) is presumptive (surgeon’s impression) and is presented as such.

### Study procedures

#### Administrative procedure

Ethical clearance was obtained from the Regional Delegation of Public Health in the Far North Region and the Institutional Review Board (IRB) of the Tokombéré District Hospital (reference No. 0112 C.E TOK2022, dated 12/12/2022). Administrative approvals were obtained from the Private Catholic Hospital of Tokombere. This was retrospective research on human subjects using existing medical records. The IRB waived the requirement for individual informed consent because there was no direct contact with patients; all data were anonymised and kept confidential, in accordance with the Declaration of Helsinki.

#### Data collection

Files of patients meeting our inclusion criteria were retained, and the following data were extracted: sex, age, presenting complaints, duration of symptoms, diagnostic methods, surgical intervention, and postoperative care and duration. We also recorded atypical presentations related to advanced age and delayed presentation. Delayed presentation was defined as presentation at our facility more than 7 days after symptom onset without prior medical care. The 7-day threshold was chosen to identify patients with markedly prolonged pre-hospital delay; it is considerably longer than the 24–48-hour thresholds commonly used in the peritonitis literature (10), and by those standards most patients in our cohort presented late. The medical records did not allow the components of delay (decision to seek care, reaching the hospital, referral, diagnosis and definitive surgery) to be distinguished; only the intervals from symptom onset to presentation and from symptom onset to surgery were available. The American Society of Anaesthesiologists (ASA) Physical Status Classification System was used to assess preoperative risk, while the Clavien-Dindo classification was used to grade postoperative complications (6, 11). ASA class was assigned preoperatively by the attending clinician and abstracted as recorded in the anaesthesia records; it was not reassigned by the investigators. All procedures were emergencies. A pre-formed online data collection form designed on Google Forms (Google, USA) (supplementary Appendix 1), was used to collect data. The form was piloted amongst five files of patients with non-traumatic gastrointestinal perforation to maintain homogeneity.

#### Data management and analysis

All the data collected was coded and stored on Google Drive in a password-protected account, and access was granted only to the investigators. The collected data were transferred to SPSS v26 (IBM, USA) for analysis.

Descriptive statistics: categorical variables were summarised as frequencies and percentages. For each variable, the denominator is the number of patients with available data for that variable; where this differs from the study population (*N* = 44), the denominator is stated in the text and in the corresponding table. For presenting symptoms, more than one of which could be recorded per patient, percentages are expressed as the proportion of patients with each symptom (denominator *N* = 44) and therefore do not sum to 100%. Continuous variables were summarised as means with standard deviations when normally distributed and as medians with interquartile ranges (IQR) otherwise.

Normality assessment: the distribution of continuous variables was assessed with the Shapiro–Wilk test and visual inspection of histograms. Age, time intervals, duration of surgery and length of hospital stay were not normally distributed and are therefore reported as medians (IQR).

Outcome definitions: the primary outcome was postoperative morbidity, graded according to the Clavien-Dindo classification from surgery until hospital discharge. Patients without any postoperative complication were classified as Grade 0, and patients who died after surgery were classified as Grade V; all operated patients (*N* = 44) were therefore included in the Clavien-Dindo analysis. The secondary outcome was postoperative in-hospital mortality, defined as death after surgery during the index admission, calculated using all operated patients (*N* = 44) as the denominator. Patients who died before surgery were excluded from the study and from the mortality calculation.

Comparative analyses: categorical variables were compared using Pearson’s Chi-square test, or Fisher’s exact test when more than 20% of cells had an expected count below 5. Continuous variables were compared between two groups using the Mann–Whitney U test and across more than two groups using the Kruskal–Wallis test. Clavien-Dindo grade was compared across age groups (paediatric vs. adult), across timing of presentation (delayed, direct, referred), and duration of surgery was compared across Clavien-Dindo grades. Given the small sample size, the small number of adverse outcomes (two postoperative deaths) and the descriptive design, no regression or other adjusted analysis of predictors of outcome was performed, as multivariable modelling would not have been statistically reliable. No formal statistical analysis plan was pre-registered; all comparative analyses are exploratory and are reported without adjustment for multiple comparisons.

Missing data: no imputation was performed. Records with missing data on both management and outcome were excluded; for the remaining records, analyses were performed on available cases, and the number of patients with available data is reported for each variable. A *p*-value < 0.05 was considered statistically significant. =

## Results

A total of 54 records on gastrointestinal perforations were identified during our study period. One was excluded for traumatic abdominal wall laceration, 4 were excluded for patients who died before the procedure, and 5 were excluded for incomplete or unexploitable data. In the end, 44 participants’ records were included in our study.

### Sociodemographic characteristics of the study population

The median age was 21.5 (IQR 9-43) years, males (*n* = 30, 68.2%) and adults (≥ 18 years, *n* = 24, 54.5%) were mostly affected (Table 1).

**Table 1:** Sociodemographic characteristics of study participants with non-traumatic gastrointestinal perforations (*N* = 44).

Variable	Category	Frequency (n)	Percentage (%)
Age (years), median (IQR)		21.5 (9–43)	
Age group	Paediatric (< 18 years)	20	45.5
	Adult (≥ 18 years)	24	54.5
Sex	Female	14	31.8
	Male	30	68.2
Occupation (adults, *n* = 24)			
	Agriculture	13	54.2
	Household	7	29.2
	Professional	1	4.2
	Student	1	4.2
	Transportation	1	4.2
	Unemployed	1	4.2

Percentages are calculated on *N* = 44, except for occupation (adults only, *n* = 24). IQR, interquartile range.

### Clinical presentation and imaging technique used

Timing of presentation was documented for 40 of the 44 patients. The majority presented directly to the hospital (within 7 days of symptom onset and without prior alternative treatment; *n* = 21/40, 52.5%), while 14 (35.0%) presented late and 5 (12.5%) were referred (Table 2); overall, delays were considerable, with a median time interval between onset of symptoms and presentation being 5 days (IQR 2.2-7). Abdominal pain (*n* = 31/44, 70.5%) was the most frequent presenting complaint, followed by fever (*n* = 16/44, 36.4%), vomiting (*n* = 10/44, 22.7%) and constipation (*n* = 9/44, 20.5%), while the most common signs were tachycardia (*n* = 28, 63.6%) and signs of generalised peritoneal irritation (*n* = 20, 45.5%).

**Table 2:** Clinical presentation and diagnostic imaging technique of study participants with gastrointestinal perforations (*N* = 44).

Variable	Category	Frequency (n)	Percentage (%)
Timing of presentation	Delayed^a^	14	35.0
	Direct	21	52.5
	Referred	5	12.5
Symptoms	Abdominal pain	31	70.5
	Fever	16	36.4
	Vomiting	10	22.7
	Constipation	9	20.5
Signs	Signs of Generalised Peritoneal Irritation	20	45.5
	Local signs of peritoneal irritation	15	34.1
	Atypical presentation related to age^b^	3	6.8
	Hypertension	3	6.8
	Hypotension	2	4.5
	Tachycardia	28	63.6
	Hyperthermia	25	56.8
ASA status	I	20	45.5
	II	6	13.6
	III	0	0.0
	IV	1	2.3
	V	17	38.6
Imaging used	Ultrasound and x-ray	16	36.4
	x-ray only	16	36.4
	Ultrasound only	11	25
	CT Scan only	1	2.3

a Presenting at our facility more than 7 days after the onset of symptoms without prior medical care. Timing of presentation was documented for 40 of 44 patients; percentages are calculated on *n* = 40. Symptom percentages are expressed as the proportion of patients with each symptom (*N* = 44; more than one symptom per patient was possible, so percentages do not sum to 100%); percentages for clinical signs, ASA status and imaging are also calculated on *N* = 44.

b Patients whose clinical picture is altered by physiologic changes associated with ageing.

ASA: American Society of Anesthesiologists physical status, as recorded preoperatively in the anaesthesia records. ASA VI (declared brain-dead organ donor) is not applicable to this cohort and has been removed from the table.

Plain abdominal x-ray only (*n* = 16, 36.4%), or an abdominal x-ray and abdominal ultrasound together (*n* = 16, 36.4%) were the main diagnostic imaging modalities**.** While the largest group of patients presented in a normal, physically fit state (ASA I, *n* = 20, 45.5%), an important proportion, *n* = 17, 38.6%, were recorded as ASA V, i.e., in a moribund state, not expected to survive without surgery. (Table 2)

### Management and outcome

The site of perforation was documented for 42 of the 44 patients (paediatric *n* = 20, adult *n* = 22). Overall, the small bowel was the most frequent site (*n* = 20/42, 47.6%), followed by the appendix, colon and stomach (*n* = 7/42, 16.7% each); one perforation (2.4%) involved another site. The most common site of perforation in the paediatric population was the small bowel (*n* = 15/20, 75.0%), and the most common site in the adult population was the stomach (*n* = 6/22, 27.3%) (Figure 1). Apart from the rectum, all other parts of the gastrointestinal tract were involved. Simple repair was the most frequently performed procedure (*n* = 29/38, 76.3%) (Table 3).

**Figure 1:**
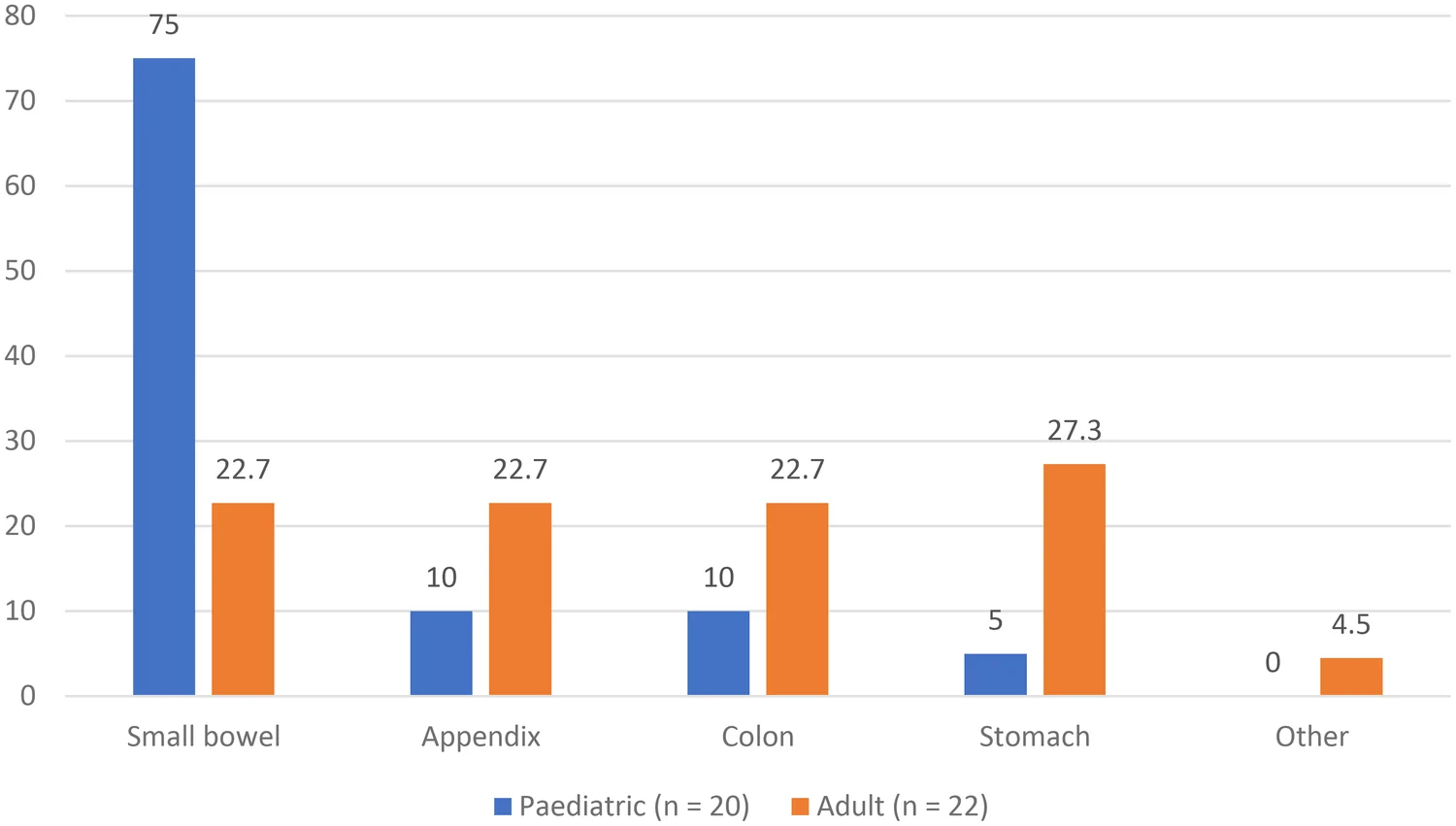
Site of non-traumatic gastrointestinal perforation by age group (*n* = 42 patients with documented site; paediatric *n* = 20, adult *n* = 22). Percentages are calculated within each age group.

**Table 3:** Intraoperative findings and operative procedures in patients managed for non-traumatic gastrointestinal perforations (*n* = 38).

Variable	Category	Frequency (n)	Percentage (%)
Intraoperative findings	Number of perforations <3	9	23.7
	Number of perforations ≥3	29	76.3
Operative procedures	Bowel resection	8	21.1
	Repair	29	76.3
	Stoma	1	2.6
	Total	38	100.0

Operative findings and procedures (Table 3) were documented for 38 of the 44 operated patients; percentages in Table 3 are calculated on *n* = 38.

Postoperative complications occurred in 27 of the 44 operated patients (61.4%), while 17 (38.6%) had an uncomplicated postoperative course (Clavien-Dindo Grade 0). Grade I was the most frequent grade (*n* = 11; 25.0% of all patients; 40.7% of those with complications) (Table 4). Two patients died after surgery (Grade V), giving a postoperative in-hospital mortality of 4.5% (2/44). Including the four patients with non-traumatic perforation who died before surgery, in-hospital mortality among the 48 patients with exploitable records who reached the hospital was 12.5% (6/48). The median duration of hospitalization was 8.5 (IQR 5.2-10) days. The median time interval between onset of symptoms and surgical intervention was 6 days (IQR 4–10; *n* = 35 patients with documented timing of presentation and interval; Table 5). The median duration of intervention was 1 h (IQR 1-3).

**Table 4:** Postoperative complications of study participants graded according to the clavien-dindo classification (*N* = 44).

Variable	Category	Frequency (n)	Percentage (%)
Clavien-Dindo classification	Grade I	11	25.0
	Grade II	6	13.6
	Grade III	3	6.8
	Grade IV	5	11.4
	Grade V (death)	2	4.5
	Grade 0 (no complication)	17	38.6
Total		44	100.0

**Table 5:** Postoperative outcome according to timing of presentation (*n* = 40).

Variable	Delayed, *n* = 14	Direct, *n* = 21	Referred, *n* = 5	Total, *n* = 40	*p*-value
Clavien-Dindo grade					
Grade 0 (no complication recorded)^b^	6 (42.9)	6 (28.6)	3 (60.0)	15 (37.5)	0.187^a^
Grade I	3 (21.4)	8 (38.1)	0 (0.0)	11 (27.5)	
Grade II	1 (7.1)	5 (23.8)	0 (0.0)	6 (15.0)	
Grade III	2 (14.3)	1 (4.8)	0 (0.0)	3 (7.5)	
Grade IV	2 (14.3)	1 (4.8)	1 (20.0)	4 (10.0)	
Grade V (death)	0 (0.0)	0 (0.0)	1 (20.0)	1 (2.5)	
Symptom onset to surgery, days, median (IQR)^c^	10 (7–12) [*n* = 11]	5 (3.8–7) [*n* = 20]	3 (2.8–5.5) [*n* = 4]	6 (4–10) [*n* = 35]	**0** **.** **009** ^d^

Values are *n* (%) unless otherwise stated.

a Fisher’s exact test; postoperative mortality by timing of presentation, Fisher’s exact test *p* = 0.125.

b No postoperative complication recorded.

c Interval documented for 35 of the 40 patients with documented timing of presentation; [n] = number of patients with data in each group.

d Kruskal–Wallis test.

There was no significant association between Clavien-Dindo grade and age group (Fisher’s exact test, *p* = 0.342) (Table 6). The duration of surgery did not differ significantly across Clavien-Dindo grades (*p* = 0.053).

**Table 6:** Clavien-dindo grade according to age group (*N* = 44).

Clavien-Dindo grade	Paediatric (< 18 y), *n* = 20	Adult (≥ 18 y), *n* = 24	Total, *N* = 44	*p*-value^a^
Grade 0 (no complication recorded)^b^	10 (50.0)	7 (29.2)	17 (38.6)	0.342
Grade I	2 (10.0)	9 (37.5)	11 (25.0)	
Grade II	3 (15.0)	3 (12.5)	6 (13.6)	
Grade III	1 (5.0)	2 (8.3)	3 (6.8)	
Grade IV	3 (15.0)	2 (8.3)	5 (11.4)	
Grade V (death)	1 (5.0)	1 (4.2)	2 (4.5)	

Values are *n* (%).

a Fisher’s exact test.

b No postoperative complication recorded.

### Outcome according to timing of presentation

Table 5 presents postoperative outcome (Clavien-Dindo Grade 0–II vs. Grade III–V, and postoperative mortality) according to timing of presentation (delayed, direct, referred) among the 40 patients with documented timing.

Among the 40 patients with documented timing, severe complications (Clavien-Dindo Grade III–V) occurred in 4 of 14 patients who presented late (28.6%), 2 of 5 referred patients (40.0%) and 2 of 21 patients who presented directly (9.5%) (Table 5). The only postoperative death with documented timing occurred in a referred patient (*p* = 0.125). The interval from symptom onset to surgery differed significantly between groups (*p* = 0.009), being longest in patients who presented late (median 10 days).

## Discussion

Since the first clinically reported case of perforated peptic ulcer in 1799, there has been a steady evolution in diagnostic and therapeutic techniques for the management of GI perforation (12). While most publications on this subject are tilted to the experience of an urban setting, research priorities in rural settings remain underserved, despite the important number of cases managed there. The median age in our study was 21.5 (IQR 9-43) years, and children (< 18 years) accounted for 45.5% of patients. This contrasts with the mean ages reported in Koutiala, Mali (34.5 years) (7) and at the Yaoundé Central Hospital, Cameroon (37.6 ± 13.5 years) (13). The male sex was seen to be predominant, accounting for 68.2% of patients, similar to the study conducted in Uganda, Mali and Cameroon (5, 7, 13, 14). Whether this reflects a true difference in susceptibility or differences in exposure and health-care use cannot be determined from our data.

### Clinical picture

More than one-third of patients with documented timing of presentation (14/40, 35.0%) presented late. This is an unfortunately common finding in LMICs and especially rural settings, as can be seen in similar studies in Mali (7) and Cameroon (6). This late patient presentation may result from a delay in seeking care, as well as a delay in accessing healthcare services once the decision has been made, as highlighted by recent studies (15). In North-West Cameroon, delays in seeking care have been attributed to fear of catastrophic health expenditure, limited awareness of the severity of symptoms and recourse to alternative treatment or self-medication, while delays in reaching care have been attributed to distance, poor road infrastructure and initial consultation at facilities not equipped for surgical emergencies (15). These factors were not measured in the present study, and their contribution in our cohort therefore remains hypothetical. We can only observe that 12.5% of patients with documented timing were referred from peripheral health facilities and that the median interval between symptom onset and presentation was 5 days.

The main presenting symptom was abdominal pain (70.5% of patients), while the most common clinical findings were signs of generalised peritoneal irritation (45.5%) (generalised abdominal pain, vomiting and constipation, board-like rigidity and signs of pneumoperitoneum). These are all findings consistent with peritoneal irritation. However, these signs were atypical or difficult to elicit in 6.8% of patients because of age-related physiological changes. The result of a gastrointestinal perforation is peritonitis, which ultimately leads to sepsis and death. A large proportion of patients (38.6%) were recorded as ASA V. This proportion is unusually high, particularly as only two patients died after surgery, and it may reflect the way the classification was applied in our setting, where ASA class was assigned by non-anaesthetist clinicians to patients with generalised peritonitis and sepsis requiring emergency surgery, rather than true moribund status. ASA grades in this cohort should therefore be interpreted with caution. Although delayed presentation is a recognised determinant of poor physiological status on admission (10, 15), the relationship between timing of presentation and ASA class was not formally tested in our cohort.

### Findings and intervention

The median time interval between onset of symptoms and surgical intervention was 6 days (IQR 4–10), much longer than the mean of 3.62 days reported by Chichom et al. in the South-West Region of Cameroon (6). One quarter of the patients in Tokombéré waited 10 days or more after the onset of their symptoms before receiving definitive surgical intervention. Possible contributing factors, which were not systematically recorded in our study, include delayed presentation (median 5 days), misdiagnosis on entry due to atypical presentations, and at other times, the unavailability of qualified medical personnel for diagnosis and intervention.

Regarding the intraoperative findings, the most common site of perforation was the small bowel (47.6%), followed by the appendix, colon and stomach (16.7% each). This anatomical distribution resembles that described in other rural African settings, where infectious and inflammatory aetiologies are frequently reported. In many Cameroonian and sub-Saharan studies, small bowel perforations—particularly those linked to typhoid ileitis—are consistently the leading cause of non-traumatic GI perforation. This is consistent with the findings of a retrospective study on non-traumatic acute peritonitis patients conducted at the National Hospital of Zinder in Niger, where the pediatric population (0-15 years) admitted for non-traumatic acute peritonitis had ileal perforations, which the authors attributed to typhoid fever (2). Typhoid intestinal perforations are feared and are a well-documented complication of Typhoid fever observed in an estimated 0.8% to 39% of untreated or improperly treated cases worldwide (3). In the present study, however, the aetiology of perforations could not be confirmed, as blood cultures, serology and histopathology were not available; any contribution of typhoid fever therefore remains presumptive. Moreover, ileal perforation is not always synonymous with typhoid perforation: in our setting, the differential diagnosis includes intestinal tuberculosis, non-specific ileal ulceration, ischaemic necrosis and perforation complicating intestinal obstruction. Although water supply, sanitation and hygiene were not assessed in this study, we can however note that Tokombéré is a rural area where subsistence farming is the main livelihood (it was also the most frequent occupation among adult patients in our cohort) and where access to potable water depends largely on seasonal streams. One could therefore suppose that these contextual factors contribute to the pattern observed, although this cannot be tested from our data alone. Children accounted for almost half of our study population (45.5%) and small bowel perforation was the predominant site in this group. Comparable observations were reported by Birkhold et al. in their scoping review (3). Savom et al. likewise reported a young population (mean age 20.3 years; 57.7% under 20 years) among patients with spontaneous terminal ileal perforation in referral hospitals in Yaoundé and Douala (5). In contrast, patients managed for non-traumatic acute abdomen of digestive origin at the Yaoundé Central Hospital were older (mean age 37.6 years) (13).

A further contrast emerges from Douala, where intestinal obstruction (32.0%) and appendicitis (24.6%) were the principal etiologies of gastrointestinal perforations (1). These discrepancies underscore the influence of age distribution and environmental context on the diagnostic profile of non-traumatic gastrointestinal perforations. Hospitals in urban centers, such as Yaoundé and Douala, serve populations with comparatively greater access to clean water and improved hygiene, which may contribute to the differing etiological patterns and anatomical sites of perforation, although this hypothesis could not be tested in our study. This may reflect the epidemiological transition in developing countries, where communicable diseases persist alongside a rising burden of non-communicable conditions, reflecting the “double burden of disease” in Cameroon’s public health landscape.

In our cohort, most patients presented with multiple perforations (≥3 perforations, *n* = 29/38, 76.3%). Typhoid commonly causes terminal ileum perforations from Peyer’s patch necrosis, producing pathognomonic characteristic oval lesions along the antimesenteric border, though other etiologies may mimic, hence the need for histopathologic confirmation even with clinical evidence (3, 14). As histopathology was not available in our setting, the multiple ileal perforations observed are compatible with, but do not prove, a typhoid aetiology. Appendiceal and gastric perforations were equally frequent. Gastric perforation was the most frequent site among adults (*n* = 6/22, 27.3%); this number is too small for any statistical comparison by age, no such test was performed, and this observation should not be interpreted as an epidemiological finding. The median duration of surgery was 1 h (IQR 1–3). A surgical intervention duration of over 2 h has been associated with a poor prognosis in other similar studies (1). The timing of presentation and of surgery, and the site and number of perforations, have been reported as determinants of outcome in other series (6, 10); however, given the small number of adverse outcomes, their independent effect could not be assessed in our cohort.

### Outcome

Postoperative complications occurred in 61.4% (27/44) of patients. Grade I complications were the most frequent (25.0%), followed by Grade II (13.6%), Grade IV (11.4%) and Grade III (6.8%); thus, 8 patients (18.2%) had a Grade III–IV complication requiring surgical, endoscopic or radiological intervention or intensive care management. These complications included surgical site infections and acute kidney injury. The postoperative in-hospital mortality rate was 4.5% (2/44); four additional patients died before surgery and were excluded from the study. Because our cohort comprises only patients who reached the hospital and underwent surgery, this figure represents postoperative in-hospital mortality among operated patients; it is subject to selection and survival bias and cannot be interpreted as the case-fatality of GIT perforation in the rural population, which is likely to be higher. Our complication rate was higher than the 31.5% reported by Chichom et al. (6), although in both series most complications were Clavien-Dindo Grade I. However, Chichom et al. reported a higher mortality rate than ours. This discrepancy may partly be explained by the difference in age; while Chichom in his cohort had older patients, our patients were younger and may have better tolerated the physiological stress of surgery. It is noteworthy that the death toll following small bowel perforation repair surgery has been on a steady decline over the last decade (3). It is a progress worth noting and should be commended and encouraged with the investments in technical equipment and staffing of health structures even in the peripheries. It, however, remains unacceptably high, with the toll being worse amongst patients with typhoid intestinal perforations (3, 6). The outcome can be attributed to a myriad of factors.

Delay in presentation and in management are some of the most important determinants of unfavourable outcomes in peritonitis patients (10, 16). A symptom duration of more than 48 h significantly increases mortality from peritonitis (16). In our cohort, timing of presentation was significantly associated with the interval from symptom onset to surgery (*p* = 0.009), whereas its association with Clavien-Dindo grade did not reach statistical significance (*p* = 0.187), possibly because of limited statistical power (Table 5). The association between delay and poor outcome has been reported by Chichom et al. (6) and attributed to local conditions of surgical practice, such as the scarcity of surgeons, the lack of appropriate diagnosis and management tools and the socio-economic conditions characterised by the total absence of social security, even for such critical and potentially deadly conditions. Ideally, better outcomes are expected in patients who present early in the course of their disease, and a prompt intervention is initiated. Preoperative shock, usually septic shock, is a well-established risk factor for mortality following intestinal perforation (16). However, the site of perforation may also alter the presentation, as signs of small bowel perforations occur sooner than those of large bowel perforations. The median duration of hospitalisation was 8.5 days (IQR 5.2–10). This timing included the time before and after the surgery. The preoperative period in this context was characterised by performing the necessary preoperative investigations. The postoperative period on the other hand, was characterised by antibiotic therapy and continuous fluid resuscitation and follow-up.

## Conclusion

This study provides a rare window into the clinical spectrum, diagnostic pathways, management strategies, and early outcomes of non-traumatic gastrointestinal tract perforations managed in a rural district hospital in Northern Cameroon. The clinical picture in this setting is characterised by delayed presentation and delayed receipt of definitive treatment (surgery). Diagnosis relied largely on clinical assessment supported by plain radiography and ultrasound, where available. Surgical intervention remained the cornerstone of treatment, with operative choice driven by perforation site and the patient’s physiologic state. These findings are descriptive, derived from a single-centre retrospective cohort of 44 surgically treated patients, and cannot be generalised. The aetiology of perforations remained presumptive. Prospective, multicentre studies including non-operated patients and systematic aetiological confirmation (blood culture, serology and histopathology) are needed.

## Study limitations

1. Single-centre, rural setting limits external validity; findings may not generalise to urban or better-resourced hospitals.
2. Potential selection bias because only patients who reached the Tokombere referral facility are included; those who died before transfer or sought care elsewhere are not captured. Patients managed non-operatively, treated in other facilities or transferred out before surgery were also not included. This introduces selection and survival bias, and the reported mortality reflects only postoperative in-hospital mortality among operated patients.
3. Incomplete or missing data in clinical records (timing of symptoms, prehospital care, laboratory results) may bias descriptive estimates and comparative analyses.
4. Limited diagnostic confirmation: reliance on clinical assessment and basic imaging means etiologies (e.g., typhoid, tuberculosis, malignancy) may be under- or misclassified without routine microbiology, histology, or CT imaging, which were altogether absent in this rural setting. Perforations were therefore described by anatomical site, and ileal perforations cannot be attributed to typhoid fever with certainty.
5. Small sample size and the small number of adverse events (two postoperative deaths and eight Clavien-Dindo Grade III–IV complications) precluded a multivariable analysis of predictors of outcome, reduced the power of the comparative analyses and increased the risk of type II error.
6. Short or incomplete follow-up may miss late complications, readmissions, or longer-term mortality, limiting assessment to in-hospital or early outcomes only.
7. Clinical heterogeneity: the cohort combined paediatric and adult patients with appendiceal, gastric, small-bowel and colonic perforations, which differ in presentation, treatment and prognosis. The sample size did not allow analyses stratified by perforation site; pooled estimates should therefore be interpreted with caution.
8. ASA class and timing of presentation were abstracted as recorded by attending clinicians; ASA class was not reassigned by the investigators, the 7-day threshold for delayed presentation was pragmatic, and the components of delay (seeking, reaching and receiving care) could not be distinguished.

## Data Availability

The raw data supporting the conclusions of this article will be made available by the authors, without undue reservation.
